# Physico-Chemical Attributes of Lemon Fruits as Affected by Growing Substrate and Rootstock

**DOI:** 10.3390/foods11162487

**Published:** 2022-08-17

**Authors:** Juan José Martínez-Nicolas, Dámaris Núñez-Gómez, Vicente Lidón, Rafael Martínez-Font, Pablo Melgarejo, Francisca Hernández, Pilar Legua

**Affiliations:** Centro de Investigación e Innovación Agroalimentaria y Agroambiental (CIAGRO-UMH), Miguel Hernandez University, Ctra. Beniel, km 3.2, 03312 Orihuela, Alicante, Spain

**Keywords:** lemon rootstock, dredged marine sediment, fruit quality, biocompounds

## Abstract

Due to its high content of bioactive compounds, the lemon is considered one of the most relevant species around the world. Its great economic importance is motivated, in addition to its fresh consumption, by its applications in the medical, pharmaceutical, and food industries, etc. However, the chemical and nutritional composition of lemon is not constant and can be influenced by external factors such as variety, weather conditions, crop management, etc. Determining the compositional variations of the fruit, essential to defining its potential use, was the main objective of this study. The physicochemical characteristics of the ‘Verna’ lemon were studied as a function of two controlled variables, the growing substrate and the rootstock. For this, 90 lemon trees were cultivated in three rootstocks and three different culture media. Lemon trees cultivated with 50% sediment/peat mix substrate presented a higher total production (590 lemons and 90.53 kg) while this production was 80% lower on trees cultivated with 75% marine sediment. *Citrus macrophylla* and *Citrus aurantium/Citrus sinensis* rootstocks showed a significantly higher production than the *Citrus aurantium*. All the fruits presented a predominantly yellow color appropriate for the market (0 < CI < +5). Nutritional and chemical parameters were consistent with data reported for the ‘Verna’ clones. All the obtained lemons were suitable for marketing and consumption both in fresh and processed forms. The results indicated the limited influence that the studied variables have on the quality parameters of lemon fruits, but they also could confirm the potential of marine sediment as a culture substrate.

## 1. Introduction

The lemon (*Citrus limon* (L.) Burm f.) is considered the third most important citrus species in the world, only behind orange and tangerine, and is socially known for its valuable nutritional, medical, pharmacological, industrial, and cosmetic properties and uses [1,2,3]. This diversity of lemon applications and uses is mainly attributed to its high content of bioactive compounds such as phenolic compounds, carotenoids, vitamins A, C and B, minerals, citric acid, and essential oils, among others [4,5,6]. Lemon juice is credited with direct health benefits due to its antimicrobial, antioxidant, antiviral, and anti-inflammatory activity [5,6,7]. However, studies indicate that the chemical composition of the lemon juice, both qualitatively and quantitatively, is not constant and can be influenced by factors such as the maturity of the fruits, lemon variety, and/or growing and management conditions [7,8,9].

Although the lemon is present in the world market, its production is restricted in areas with warm and temperate climates, because they are sensitive to low temperatures. Among the most important countries, Spain is the main lemon producer in the Mediterranean region with 42% of the total European production followed by Italy (38%), Greece (5%), and Portugal (1.6%) among other minority countries [10]. Due to the climatic specifications of lemon trees, in Spain, its production is concentrated on the southeast region, with emphasis on the south of the Valencian Community, the Region of Murcia, and some areas of Almería [1].

Among all the varieties of lemons, the ‘Verna’ variety is the second most relevant in the Valencian Community behind the ‘Fino’ variety. The main characteristic of this lemon variety is that, in general, they present an abundant harvest with medium/big sized fruits, a good acidity of its juice, and few seeds. Another advantage is that the fruits do not present a loss of quality over time, which allows for a staggered harvest [11,12].

As the variety choice based on productive and fruit quality criteria is a relevant decision for lemon cultivation, the rootstock selection also is fundamental due to its impact on crop management, since the rootstock can present greater resistance to diseases and/or different adaptation capacities to edaphoclimatic conditions. In this sense, *Citrus macrophylla* and *Citrus aurantium* are the most common rootstocks used in southeast Spain for lemon cultivation. *Citrus macrophylla* is the most demanded rootstock used for lemon trees, mainly due to its vigor or speed of growth; that is, the trees with *Citrus macrophylla* rootstocks reach a larger size in less time, which can translate into great productivity. However, when grafted with ‘Verna’, it can produce oversized lemons. *Citrus aurantium* is already the traditional lemon rootstock, and although its use gives the tree great resistance to hydro edaphic environments, its affinity with the ‘Verna’ variety is limited and therefore its commercial and productive life are also limited [11]. Intermediate wood such as *Citrus aurantium/Citrus sinensis* is commonly used both to avoid possible incompatibilities between rootstock and variety, as well as to improve the quality of the fruits.

On the other hand, different studies confirm the influence of the culture medium on the quality of the fruit (productive yield, morphological characteristics, sugar content, antioxidant content, etc.), mainly due to the adaptation/resistance of the trees to the different parameters of the substrate, such as pH (which determines the mobility and availability of microelements), conductivity (which indicates the accumulation of salts in the substrates), and porosity (oxygen availability), among others [13,14,15,16]. Historically, peat has been and continues to be the primary substrate used in agriculture. However, its high demand worldwide—as well as the increasingly high costs of its extraction, storage, and transport—highlight the need to find alternative substrates with good compatibility with plants, that is, good vegetative development, high production, and fruit quality, among others. Thus, in a circular economy and sustainable context, the study of alternative crop media has increased in recent years; among them, many are based on waste, such as port sediment [17,18,19,20,21,22,23]. Although numerous works confirm the potential use of marine sediments as substrates for food and ornamental crops, no studies on lemon cultivation have been found, being one of the most important worldwide [23,24,25,26,27].

In this context, the objectives of this work were: (a) evaluate the potential use of phytoremediated marine sediment as an agricultural substrate for lemon cultivation; (b) define the influence of the cultivation substrate on the morphological and quality characteristics of lemons, with emphasis on their biocomposites; and (c) identify the potential variations/adaptations of the physicochemical composition of lemons related to the most common commercial rootstocks used in southeastern Spain.

## 2. Materials and Methods

### 2.1. Plant Material and Experimental Design

In this work, the rootstock/substrate impact on lemon fruit characteristics (*Citrus limon* (L.) Burm var. ‘Verna’) was studied by using nine different treatments (substrate/rootstock combinations) as shown in Table 1. The treatments were differentiated by two controlled variables: the rootstock (*n* = 3) and the culture media (*n* = 3). The most common rootstocks used in commercial lemon cultivation were: (i) *Citrus macrophylla*; (ii) *Citrus aurantium*; and (iii) a combination between *Citrus aurantium* and *Citrus sinensis,* usually called ‘sweet orange intermediate wood’. Related to the grown media, three substrates composed of peat and phytoremediated marine sediment in different proportions were evaluated: (i) 25% sediment + 75% peat; (ii) 50% sediment + 50% peat; and (iii) 75% sediment + 25% peat. The marine sediment used in this work came from Livorno port (Italy), it was previously phytoremediated for three years and has already been used successfully in other ornamental and food crops [21,23,25,26,28].

Briefly, the cultivation soil was carefully prepared by mixing peat and the phytoremediated sediment according to the specified percentages (Table 1). Once the culture substrates were prepared, the lemon trees purchased from a commercial nursery—supplied in plastic containers with coconut fiber with a capacity of 1 L, and with a trunk diameter of less than 1 cm—were planted in polypropylene pots with a 40 L maximum capacity. Once planted in the containers, all the plants were stripped and cut to the same height (0.75 cm), aiming to standardize all treatments and lemon trees according to the commercial management of new plantations in the Spanish southeast, the main lemon growing area in Spain.

As highlighted above, the present study considered 9 different treatments (1 lemon variety × 3 rootstocks × 3 substrates). For each treatment (*n* = 9), a total of 10 trees were evaluated with a random distribution experimental design by blocks (*n* = 5) and two trees per block (*n* = 2). In total, 2-year-old 90 lemon trees (3 substrates × 3 rootstocks × 5 blocks × 2 trees per block), grown in polypropylene pots, were evaluated in an experimental plot of Miguel Hernandez University (Orihuela, Spain) located in the Southeast of Spain (38°04′ N, 0°58′ W, 26 m above sea level). The average annual temperature was 19 °C, with moderate winters (minimum of 11 °C in January) and warm summers (maximum of 28 °C in August). The mean annual precipitation was 300 mm, and most of this precipitation was recorded in the spring and autumn seasons. The area presented high insolation, with approximately 3000 h of sunshine per year. The highest sunshine percentage was registered during the summer months (more than 352 h in July) and the lowest in December and January [29].

Fertigation water was supplied through a drip irrigation system, with one pipeline for each differentiated treatment based on the content of the marine substrate (25%, 50%, and 75%), with one drip per tree (4 L h^−1^). In total, during the entire two-year experiment, an average of 3.12 m^3^ of water per tree was applied. Both the cultivation conditions and the crop management were kept homogeneous throughout the trial to minimize external influences and to study the morphological and nutritional lemon variations/differences objectively.

Related to plant material, the ‘Verna’ variety of lemon fruits are considered spring/summer lemons, with the main harvest between March and September. The main bloom of the ‘Verna’ variety begins in April–May, but it shows several continuous blooms during the year. In general, up to five or six blooms can be distinguished during the year, from which three commercial harvests are obtained, these being: (i) the main harvest (March to June); (ii) the second lemon harvest (June), and (iii) the ‘*rodrejo*’ harvest (between August and October), depending on weather conditions. In this study, the lemons evaluated correspond to the main bloom.

In all cases, the lemon fruits were harvested manually once the fruit reached commercial maturity (equatorial caliber > 45 mm and yellow peel color) in accordance with the provisions of the marketing standard for citrus fruits [30]. The production evaluation was carried out in-situ considering both the number of fruits per tree and the total weight per tree (EM-60KAL, Cobos precision). After collection, the lemon fruits were immediately transported to the laboratory for their processing and characterization.

### 2.2. Morphological Characterization

The morphological characterization was carried out on all the fruits collected for each treatment (*n* = 9). The fruit weight (FW, g) was determined with an analytical balance with a precision of 0.02 g (GramPrecision, Series BH-1200; Barcelona, Spain). Measurements of equatorial diameter (ED, mm), fruit length (FH, mm), mamelon length (ML, mm), and peel thickness (PT, mm) were evaluated using a digital caliper (model 500-197-20, 150 mm; Mitutoyo Corp., Aurora, IL, USA). Additionally, the number of seeds and carpels was also monitored by simple visual and manual counting. All measurements were made at constant room temperature (22 ± 2 °C). The results presented are the mean values (91 ≤ *n* ≤ 590) with the standard deviation in parentheses.

### 2.3. Internal and External Color Determination

For the external and internal color measurement a digital colorimeter (model CR-300, Minolta, Osaka, Japan) was used. For the external color, four equidistant peel measurements were made in the equatorial zone of the fruit, while the internal color was studied through the juice color obtained as specified in the chemical and nutritional characterization section of this work. All measurements were performed at constant room temperature (22 ± 2 °C). The color was evaluated according to the Commission Internationale de l’Éclairage (CIE) [31] and expressed as L*, a*, and b* parameters to indicate the lightness of the color, its position between green and red, and its position between blue and yellow, respectively [32]. Additionally, the target color $\left(C^{*}=\sqrt{\frac{a^{*}2+b^{*}2}{2}}\right)$; Hue angle $\left(H^{o}=\tan^{-1}\frac{b^{*}}{a^{*}}\right)$; and color index $\left(CI=\frac{a^{*}\times 1000}{L^{*}\times b^{*}}\right)\;$ were also calculated. The results correspond to the mean values with the standard deviation in parentheses.

### 2.4. Chemical and Nutritional Characterization

Chemical analyses were performed using five juice samples for each treatment (*n* = 9). Lemon juice was carefully obtained using a commercial manual juicer (Citromatric Deluxe, MPZ-22, Braun GmbH, Kronberg, Germany) at constant room temperature (22 ± 2 °C). Immediately after being squeezed, the juice was characterized for color, as specified in the previous section, and pH, titratable acidity (TA, g acid citric L^−1^) and total soluble solids (TSS, °Brix) parameters following the methodology described by Aguilar-Hernández et al. [9]. The ratio $\frac{TSS}{TA}$, or maturity index (MI), was also calculated. Additionally, the juice yield (JY, % (w:w)) was also determined for each sample using an analytical balance with a precision of 0.02 g (GramPrecision, Series BH-1200; Barcelona, Spain) aiming to identify significant impacts on relevant commercial parameters, since the technical standard for the commercialization of citrus fruits establishes a minimum juice content of 20% as a requirement [30]. The results presented correspond to the mean values with its standard deviation in parentheses.

#### 2.4.1. Antioxidant Activity and Total Phenolic Content

The antioxidant activity (AA) of lemon juice was studied using five juice samples per treatment (Table 1) following the methodology described by Aguilar-Hernández et al. [9] for three different and consolidated methods, these being: (i) 2.2′-radical method Azinobis [3-ethylbenzothiazolin-6-sulfonic] (ABTS^+•^); (ii) radical method 2.2′-Diphenyl-1-Picrylhydrazyl (DPPH^•^); and (iii) reduction of the ferric ion (FRAP) by means of a UV–visible spectrophotometer (Termospectromic Helios Gamma model, UVG 1002E, Cambridge, UK). The results correspond to the mean values (*n* = 5) with its standard deviation and are expressed in mmol Trolox per liter of juice. The total phenolic content (TPC) was also determined using the Folin–Ciocalteu colorimetric reagent as described by Singleton et al. [33]. The measurements were made by spectrophotometry (Termospectromic Helios Gamma model, UVG 1002E, Cambridge, UK) and concentrations were calculated against the gallic acid calibration curve. The results are shown in mg equivalents of gallic acid (GAE) per 100 mL of juice and the values correspond to the mean values (*n* = 5).

#### 2.4.2. Sugars and Organic Acids Content

The quantification of sugars and organic acids was performed by Agilent 1100 high-performance liquid chromatography (HPLC) with ChemStation software by installing a Supelcogel C610H column, 30 cm × 7.8 mm; and a pre-column Supelguard, 5 cm × 4.6 mm (Supelco, Bellefonte, PA, USA). For the organic acids measurement, a diode array detector (DAD) (Diode Aray DAD G1315A) set at 210 nm was used, while a refractive index detector (RID) (G-1362-A) was employed for sugar content determination. Reference standards were used for organic acids (L-ascorbic acid, malic acid, citric acid, oxalic acid, acetic acid, lactic acid, and succinic acid) and sugars (glucose, fructose, and sucrose) supplied by Sigma Aldrich (Poole, Dorsert, UK) with calibration curves with R^2^ ≥ 0.999. The results obtained correspond to the mean values (=5) and are expressed in g 100 mL^−1^.

### 2.5. Statistical Analysis

The experimental data obtained were evaluated by means of a one-way analysis of variance (ANOVA) followed by a mean separation contrast using the Tukey HSD test for *p* < 0.05. In addition, principal component analysis (PCA) and cluster analysis (CA) were also performed. Cluster analysis was applied to the standardized data for hierarchical associations using Ward’s method for clustering and squared Euclidean distance as a measure of dissimilarity. Statistical analyses were performed using Statgraphics software (Centurion 18, Statgraphics Technologies, Inc., The Plains, VA, USA).

## 3. Results and Discussion

Due to the large number of experimental data and the possibility of multiple combinations of the variables studied, the results are presented differently by the substrate (*n* = 3), rootstock (*n* = 3) and, additionally, by treatment (*n* = 9) aiming to identify and define the impact, differences, and/or similarities of the parameters evaluated.

Related to the lemon production (Table 2), the results showed that the trees cultivated with 50% sediment and peat substrate presented higher production, both number of fruits (19.66 fruits) and yield (3.12 kg of lemons per tree) and, therefore, a higher total production (590 lemons and 90.53 kg). These values are significantly different when compared to the production obtained in the lemon trees cultivated with the other substrates, but with emphasis on the substrate with a higher sediment content (75%). In this case, the production in number of fruits and weight per tree was 79% and 80% lower, respectively. Analyzing production results related to the rootstocks used, these differences were diluted and no significant differences were observed between *C. macrophylla* and *C. aurantium/C. sinensis* rootstocks, which presented more than 14 lemons per tree and 2.28 kg per tree. However, both rootstocks were significantly superior when compared with *Citrus aurantium* rootstock results, where its production values were only 3.03 fruits and 0.65 kg per tree, respectively. The results indicate a substrate influence on the production obtained; likewise, the *Citrus macrophylla* and *Citrus aurantium/Citrus sinensis* rootstocks showed a significantly higher production than the *Citrus aurantium* (Table 2). The results agree with the bibliography, where—in non-limiting water conditions—a greater vigor of *Citrus macrophylla* rootstock has been reported, inducing a higher vegetative growth and higher production compared to *C. aurantium* [34].

Although some authors attribute these productive differences between the rootstocks to their differential capacity to absorb water and nutrients due to the physical differences between the root systems [35,36], the tree age (2-year-old), and the conditions and characteristics of the crop media should not be forgotten, so additional studies to verify the real impact of the rootstock and/or substrate on production need to be carried out.

### 3.1. Morphological Characterization of Lemon Fruits

The lemon fruits’ morphological characteristics showed few significant differences, both when compared the results related to the substrate and related to the rootstock (Table 3). In this sense, the lemon fruits grown with the 25% sediment and 75% peat substrate presented the highest fruit weight (168.15 g) and mamelon length (15.24 mm) but the smallest equatorial diameter (63.10 mm); while that the lemons fruits obtained with a substrate with higher amounts of marine sediment (50% and 75%) did not show significant differences between them. For all cases, no significant differences were observed for peel thickness (≥6.73 mm), number of carpels (≥8.92), number of seeds (≥1.31), and juice yield (27%) parameters, which could indicate the limited influence of the cultivation substrate in the morphological characteristics of lemon fruits.

Related to the rootstock type used, the *Citrus aurantium* fruits presented a greater weight (176.13 g), equatorial diameter (64.07 mm), and fruit length (98.05 mm), configuring the most characteristic morphology of ‘Verna’ lemons [12]. In addition, they also presented the highest number of seeds per fruit (2.31). In the opposite site, *Citrus macrophylla* lemons presented a shorter mamelon length (15.01 mm). Again, the results for peel thickness, number of carpels, and juice yield parameters did not present differences between the rootstocks.

In addition, considering the fruit size as a fundamental parameter for its market valorization, it should be noted that—in all cases—the lemon fruits obtained presented a medium/large size, so they would be suitable for fresh consumption and, therefore, its acceptance in the market. This decrease in the weight of cultivated plants/fruits with high contents of marine sediments was already observed and confirmed for lettuce and strawberry [25,26]. Thus, some studied treatments could present commercial competitive benefits related to the lower caliber of ‘Verna’ lemons on *Citrus aurantium/Citrus sinensis rootstock* and with 75% marine; since ‘Verna’ lemons tend to present excessive fattening, mainly in rainy springs, exceeding their commercial size and, therefore, losing their economic value.

The lemon fruits reported in the bibliography for different varieties (‘Eureka’ and ‘Fino’) and grafted on other rootstocks (*Citrus volkamerina*) presented both smaller equatorial diameter and fruit weight, and in some cases, bigger peel thickness [37,38,39,40]. In this sense, the results could confirm, from a morphological characteristic point of view, the adequacy of the marine sediment in different mixes with peat as a growing substrate applicable to lemon cultivation, but it also shows the ability of the rootstocks to adapt to the conditions of the growing medium.

### 3.2. Internal and External Color

The external color of the fruit is shown to be one of the most relevant quality parameters in consumer acceptance, therefore determining variations and/or modifications in color is relevant both at an agronomic and economic level [41]. In this sense, the external color and the juice color were determined for all the treatments studied, and their results are presented in Table 4.

Related to the peel color, the less luminous lemons (L*) were identified for the fruits obtained with the substrate with 25% sediment (70.12) and for the *Citrus macrophylla* lemons (70.80); the other treatments did not present significant differences for luminosity values (<71). All the fruits, regardless of the substrate and/or rootstock variable, showed greenish-yellow colorations as indicated by the positive values of a* and b*, respectively. The parameters C* and H° were not affected by the rootstock, but presented a significant difference between the substrates used, where the lemon fruits obtained with the substrate with 25% sediment presented the lowest values of C* (53.32) and higher values of H° (84.92). In general, all the fruits presented a predominantly yellow color appropriate for market (0 < CI < +5) [12,42].

Like the external color of the fruits, the juice color results were quite homogeneous. Thus, in this case, the least luminous juice (L*) was obtained with the lemons grown with the substrate with 75% sediment (41.21). Only the *C. aurantium* lemon juice presented a reddish coloration (positive value of a*) compared to the other treatments with greenish chlorination (negative values of a*). All the juices presented yellowish coloration (positive b* values) being: (i) by substrate 75% > 50% > 25%; and (ii) by rootstock *C. aurantium* > *C. macrophylla* > *Citrus aurantium/Citrus sinensis.* In relation to the CI, all the juices presented yellowish green tones with the most intense values for the fruits with 25% sediment (−8.85) and *C. aurantium* as standard (7.49).

The results obtained are consistent with the literature, since other authors have already indicated a limited rootstock influence on the fruit color (internal and/or external), being much more influenced by other factors—such as variety, temperature, solar incidence and humidity, among others [9,43,44,45]—which would be affected by the conditions of the culture substrate.

### 3.3. Chemical Characterization

In order to determine the potential of the remediated marine sediment as an agricultural substrate applied to lemon cultivation and its interaction with the rootstock, it was necessary to determine the quality parameters of the juice obtained since the variations in the biocompounds content could be a relevant factor for decision making. The results are shown in Table 5.

The highest pH values (3.04) were identified for lemons cultivated with 75% sediment and those obtained with *Citrus aurantium/Citrus sinensis* rootstock; while for the other treatments the values remained between 2.49 and 2.54 without significant differences. The lemons obtained with 25% sediment presented the lowest values of total soluble solids (8.96 °Brix), the other treatments did not present significant differences and their values were around 9 °Brix. These results are higher than the data reported on the bibliography for ‘Verna’ variety (normally between 6 and 7.5 °Brix), but similar to those determined for ‘Fino’ variety [34,46].

On the other hand, TA showed a similar trend for the two studied variables, with significant differences both as a function of the substrate and the rootstock. In this sense, the lemon cultivated with 75% sediment showed the highest TA values (64.32 g citric acid L^−1^) followed by 50% sediment and 25% sediment, with 62.79 and 59.71 g citric acid L^−1^, respectively. Depending on the rootstock, *Citrus aurantium/Citrus sinensis* lemons presented the highest values (63.88 g citric acid L^−1^) followed by *C. macrophylla* (62.33 g citric acid L^−1^) > *C. aurantium* (59.75 g citric acid L^−1^). The results, although a bit higher than those reported in the bibliography, could be considered within the expected range for lemon juice [9,47]. The MI for all treatments studied was between 1.41 and 1.52 (Table 5). Finally, the juice yield was similar for all the treatments studied (rootstock and substrate) with increasing values from 24% to 27% (w:w). These results are consistent with data reported for the ‘Verna-62’ and ‘Verna-50-2’ clones [46].

### 3.4. Antioxidant Activity and Total Phenolic Content

The health benefits of lemon fruit have been maintained throughout generations mainly due to its antioxidant activity; based on this, the total phenolic content (TPC) and antioxidant activity—evaluated by DPPH^•^, ABTS^+•^, and FRAP methods—were determined for all the treatments and the results are shown in Table 6.

When analyzing the results according to the substrate, significant differences were only observed for FRAP method, where lemons grown with 75% sediment presented the highest values (1.25 mmol Trolox L^−1^) while with 25% sediment barely reached 1.08 mmol Trolox L^−1^. The results agree with those reported for ‘Verna’ lemon, but are different from those defined for other ‘Verna’ clones [9,46]. These differences can be able to indicate the variety influence in this parameter, but also could be attributed to the to the time of fruit harvest [48]. On the contrary, when analyzing the results according to the rootstock used, significant differences were observed for FRAP, DPPH, and TPC. *Citrus aurantium/Citrus sinensis* lemons showed the lowest result for DPPH (2.91 mmol Trolox L^−1^) but the highest iron oxidation capacity FRAP (1.25 mmol Trolox L^−1^). Czech et al. [49] indicated that the highest antioxidant activity in the critic fruits generally is in the peel, mainly due to the tannins’ compounds. In that study, the authors evaluated a total of nine citric fruits (orange, grapefruit, mandarin, lime, etc.), and the highest antioxidant activity was observed for mandarin peel (218.1 and 444.8 mmol Trolox g^−1^ determined with ABTS and DPPH, respectively).

The highest TPC, significantly different from the other rootstocks, was determined for *Citrus aurantium* lemons (104 mg GAE 100 mL^−1^), which could indicate a lower degree of maturity compared to the fruits of the other rootstocks [50]. Xi et al. [51] determined the phenolic profile of different lemon fruit parts; as in the previous study, the authors indicated that lemon juice had the lowest phenolic content, being 87% and 80% lower than the peel and pulp, respectively.

### 3.5. Sugars and Organic Acids

As indispensable contributors to fruit quality, organic acids and soluble sugars can affect fruit flavor and indirectly exert beneficial and disadvantageous effects on marketable fruit quality [52]. In this study and for all juice samples, two sugars, glucose and fructose; and 4 organic acids, citric, malic, ascorbic, and succinic acid were identified (Table 7).

For all the lemon fruits, glucose was the majority sugar (≥0.75 g 100 mL^−1^) followed by fructose (≥0.51 g 100 mL^−1^), and no significant differences between the variables analyzed (rootstock or substrate) were observed. The predominance of glucose against fructose confirms what was established for “acidic” citrus varieties, in contrast to “non-acidic” varieties where fructose predominates against glucose [3,9,53,54]. The sugar content determined was significantly lower than those reported for lemons grown in soil, regardless of the variety and/or rootstock used, which would indicate the influence of the substrate, or its limitation since was a pot crop, on the chemical fruit characteristics. In this sense, Aguilar-Hernández et al. [9] reported values between 2.2–3.1 g 100 mL^−1^ and 2.7–4.0 g 100 mL^−1^ for glucose and fructose, respectively, for ‘Fino’ and ‘Verna’ clones but cultivated soil directly.

The main organic acid quantified in the juices was citric acid followed by succinic acid > malic acid > ascorbic acid, which coincides with what was previously reported [3,9,55]. In all cases, no significant differences were observed depending on the variables studied (substrate and rootstock), which could indicate the limited impact of the variables on the relevant metabolic pathways. The ascorbic acid content was significantly lower than that reported for other citrus fruits such as clementine (values between 1.32 and 1.58 g 100 mL^−1^) and grapefruit (values between 4.11 and 0.23 g 100 mL^−1^), but with higher values of malic and citric acid than those determined for the sweet orange, tangerine, and sour orange cultivated in Turkey [56,57,58,59]. Considering that citric acid is the most important food acidulant, among other industrial applications [57], the high citric acid content of the lemons obtained in this study would indicate the fruit commercialization potential (fresh and/or processed) and therefore, would confirm the potential of marine sediment as a culture substrate.

### 3.6. Treatment Difference Identification

**Figure 1:**
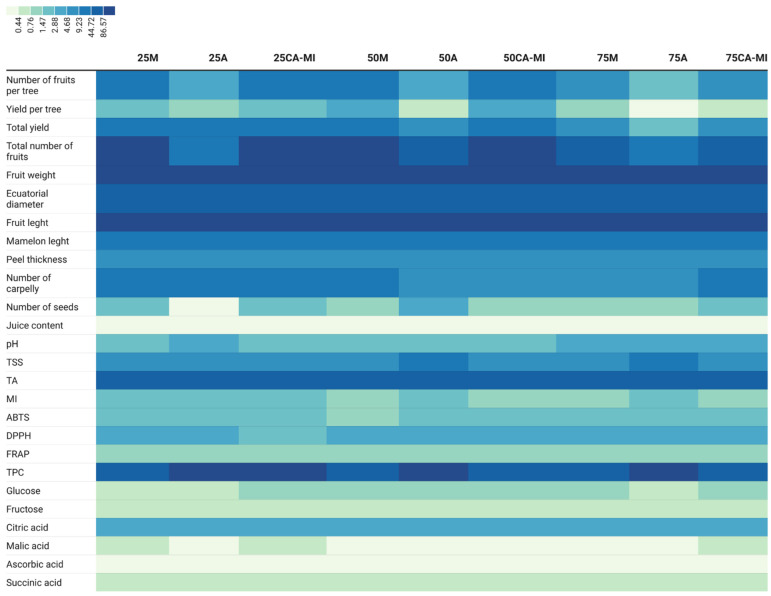
Differential heatmap of the morphological characteristics and chemical parameters evaluated in ‘Verna’ lemons depending on the substrate, where 25, 50, and 75 represent the content of marine sediment—being 25%, 50% and 75%, respectively; and the rootstock used, where CM, CA-MI, and CA represent the *Citrus macrophylla rootstock Citrus aurantium/Citrus sinensis,* and *Citrus aurantium,* respectively (Table 1).

More specifically, the results were analyzed individually for each of the nine treatments (one variety × three substrates × three rootstocks) in order to identify the differences and/or similarities between them in more detail. The results indicated differences mainly related to production, number of seeds, total phenolic content, and malic acid, while the rest of the morphological parameters presented homogeneity of the results between the samples (Figure 1). Since the climatic and crop management conditions were homogeneous in all the samples, in addition to being the same lemon variety, based on the results, the limited impact of the substrate and rootstock on the morpho-chemical characteristics of the lemons could be confirmed.

### 3.7. Principal Component Analysis (PCA)

To achieve a better understanding of the trends and relationships between the parameters studied (37) for the variables used (2), principal component analysis (PCA) was applied, considering the analysis based on the type of substrate used (with different content of marine sediment at 25%, 50%, and 75%) and rootstock (*Citrus macrophylla, Citrus aurantium,* and *Citrus aurantium/Citrus sinensis*).

**Figure 2:**
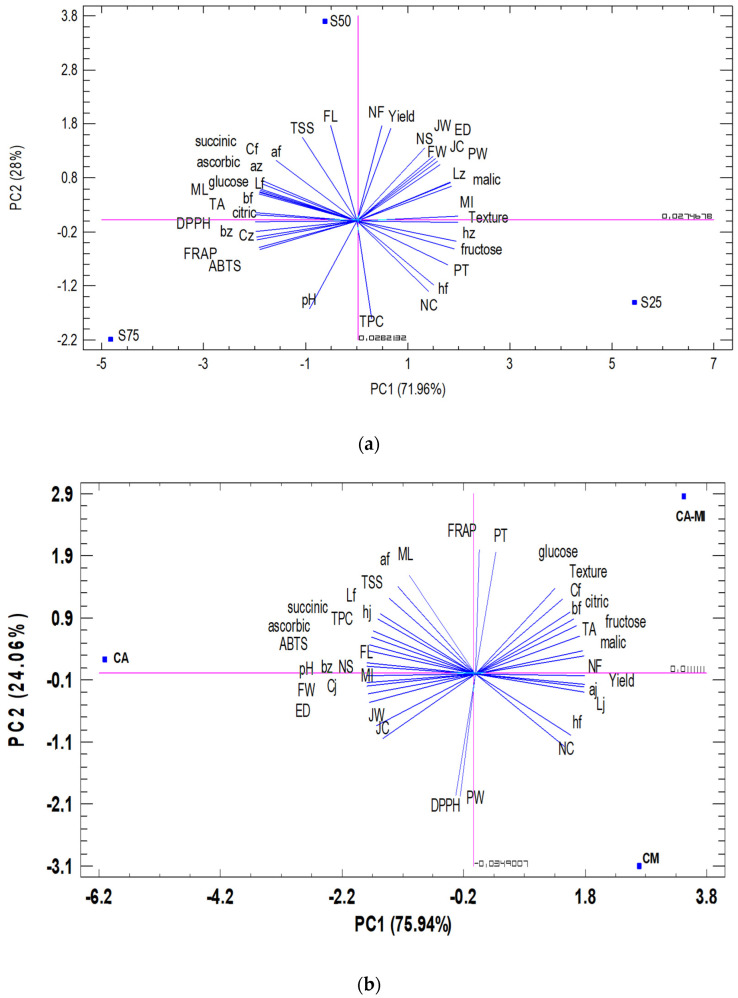
Principal component analysis of the morphological characteristics and chemical parameters evaluated in ‘Verna’ lemons depending on the (**a**) substrate where S25, S50, and S75 represent the content in the marine sediment substrate—being 25%, 50%, and 75%, respectively; and (**b**) rootstock used, where CM, CA-MI, and CA represent the *Citrus macrophylla* rootstock. *Citrus aurantium/Citrus sinensis*, and *Citrus aurantium*, respectively.

In this sense, depending on the type of culture substrate, the first two main components (PC) explained 100% of the total variation. The first component (PC1), which represents 71.96% of the total variance, was related to the variables analyzed: mamelon length, equatorial diameter, peel weight (PW), TA, MI, ABTS, DPPH, FRAP, the fruit color parameters (L*, b*, C*) and juice color parameters (L*, a*, b*, C*, H°), citric acid, malic ascorbic acid, succinic acid, glucose, and fructose (Figure 2a). These variables are related with the color of the fruit and juice, organic acids, and sugars. On the other hand, PC2 represented 28% of the total variance. It was correlated with: number of fruits, production, fruit weight, equatorial diameter, fruit length, number of carpels, number of seeds, juice content, pH, TSS, TPC, and the fruit color parameters a* and H° (Figure 2a). In short, it describes the production, fruit form, amount of juice, and TPC.

**Figure 3:**
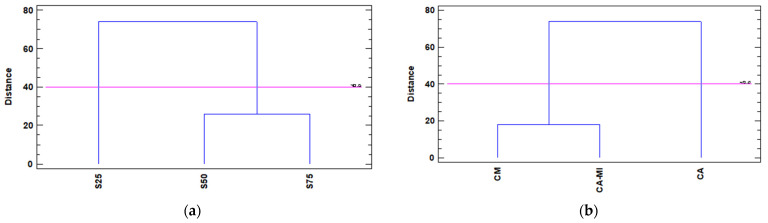
Dendrogram for the cultivation of ‘Verna’ lemons using Ward’s method based on the squared Euclidean distance of the morphological and chemical parameters, depending on the type of (**a**) cultivation substrate, where S25, S50, and S75 represent the content in the marine sediment substrate—being 25%, 50%, and 75%, respectively; and (**b**) rootstocks used, where CM, CA-MI and CA correspond to the *Citrus macrophylla* rootstocks *Citrus aurantium/Citrus sinensis*, and *Citrus aurantium*, respectively.

The PCA results for the type of substrate showed that the PC1 axis allows to discriminate between the three substrates (25%, 50%, and 75%) and PC2 discriminates the substrate with 50% sediment from the other two (25% and 75%). The cluster analysis indicates that 50% and 75% are close to each other and far from 25% (Figure 3a).

On the other hand, the statistical analysis based on the rootstock used again indicates that the first two main components (PC) explained 100% of the total variation (Figure 2b). In this case, the first component (PC1)—which represents 75.94% of the total variance—was related to the variables analyzed: number of fruits; production; fruit weight; equatorial diameter; fruit length; number of seeds; yield of juice; pH; TA; ABTS; TPC; the color parameters L*, b*, C*, and H° for the peel and L*, a*, b*, C*, H° for the juice color; citric acid; malic acid; ascorbic acid; succinic acid; and fructose. These variables are related directly with the production, shape of the fruit, fruit and juice color, organic acids, and fructose. PC2 represented 24.06% of the total variance and was correlated with mamelon length; peel thickness; number of carpels; juice content; peel weight (PW); TSS; FRAP; DPPH; and the color parameters L*, a*, C*, and H° from the fruit. It basically describes the production, shape, and color of the fruit and antioxidant activity.

The PCA results for rootstock type showed that the PC1 axis allows *Citrus aurantium* to be discriminated from *Citrus macrophylla* and *Citrus aurantium/Citrus sinensis*; and PC2 discriminates between the three combinations (*Citrus macrophylla, Citrus aurantium*, and *Citrus aurantium/Citrus sinensis)*. The cluster analysis indicates that the rootstocks *Citrus macrophylla* and *Citrus aurantium/Citrus sinensis* are close to each other and far from *Citrus aurantium* (Figure 3b).

## 4. Conclusions

The results of the present study in relation to the vegetative development of the plants, as well as the productivity obtained from 2-year-old lemon trees, indicated the potential of the phytoremediated marine sediment mixed with other substrates as an agricultural substrate applied to the cultivation of lemon. Indeed, the substrate composed of a 50%/50% mixture of marine sediment and peat presented the highest fruit production (both in number of fruits and in weight), while the *Citrus macrophylla* and *Citrus aurantium/Citrus sinensis* rootstocks were the ones with the highest lemon fruit production regardless of the growing medium.

The impact of marine sediment on the reduction of the fruit size/weight could be used as a tool to control excessive fruit development in some lemon varieties such as ‘Verna’.

The lemons obtained in the nine substrate/rootstock combinations studied presented appropriate characteristics for their categorization as commercial. The nutritional value of the ‘Verna’ lemon juices was very homogeneous among the treatments studied; that is, the limited influence of both the rootstock and the culture substrate on the chemical characteristics of the fruits would be confirmed.

## Figures and Tables

**Table 1:** Specifications of the rootstock and the crop media of the *Citrus limon* ‘Verna’ var. lemons evaluated in this study, with emphasis on the acronym used.

Rootstock	Grown Media	Acronym
*Citrus macrophylla*	75% peat + 25% sediment	25 M
*Citrus macrophylla*	50% peat + 50% sediment	50 M
*Citrus macrophylla*	25% peat + 75% sediment	75 M
*Citrus aurantium*	75% peat + 25% sediment	25 A
*Citrus aurantium*	50% peat + 50% sediment	50 A
*Citrus aurantium*	25% peat + 75% sediment	75 A
*Citrus aurantium/Citrus sinensis*	75% peat + 25% sediment	25 CA-MI
*Citrus aurantium/Citrus sinensis*	50% peat + 50% sediment	50 CA-MI
*Citrus aurantium/Citrus sinensis*	25% peat + 75% sediment	75 CA-MI

**Table 2:** Variation of the production of ‘Verna’ lemons obtained depending on the type of substrate and the rootstock used. The values per tree correspond to the mean with its standard deviation in parentheses. Total values are the sum of production per independent variable.

	No. of Fruits/Tree	Yield (kg Tree^−1^)	Total No. of Fruits	Total Yield (kg)
**Type of substrate (sediment content, %)**				
25%	9.63 (9.75) ^c^	1.68 (1.59) ^a^	289 ^c^	50.51 ^b^
50%	19.66 (15.73) ^a^	3.12 (2.21) ^b^	590 ^b^	90.53 ^a^
75%	4.13 (7.13) ^b^	0.58 (0.96) ^c^	124 ^a^	17.28 ^c^
**Type of rootstock**				
*Citrus macrophylla*	14.83 (13.45) ^a^	2.35 (2.09) ^a^	445 ^b^	70.78 ^b^
*Citrus aurantium*	3.03 (4.84) ^b^	0.65 (0.98) ^b^	91 ^a^	18.86 ^a^
*Citrus aurantium/Citrus sinensis*	15.56 (14.67) ^a^	2.28 (2.06) ^a^	467 ^c^	68.67 ^c^

The different letters within the columns for each independent variable (type of substrate and rootstock) indicate significant differences according to the Tukey HSD test (*p* < 0.05).

**Table 3:** Morphological characteristics of the ‘Verna’ lemons obtained depending on the type of substrate and the rootstock used. The results correspond to the mean values with their standard deviation in parentheses.

	Fruit Weight (g)	Equatorial Diameter (mm)	Fruit Length (mm)	Mamelon Length (mm)	Peel Thickness (mm)	Number of Carpelly	Number of Seeds	Juice Content (w:w)
**Substrate (sediment content, %)**								
25%	168.15 (48.80) ^a^	63.10 (6.16) ^a^	93.66 (12.54) ^a^	15.24 (4.83) ^a^	7.08 (1.49) ^a^	9.31 (1.19) ^a^	1.59 (0.91) ^a^	0.27 (0.07) ^a^
50%	168.91 (34.78) ^b^	63.32 (4.47) ^b^	96.39 (10.02) ^ab^	16.18 (4.20) ^ab^	6.73 (1.30) ^a^	8.92 (1.59) ^a^	1.66 (1.74) ^a^	0.27 (0.07) ^a^
75%	142.42 (33.00) ^b^	59.78 (4.59) ^b^	94.10 (10.13) ^b^	16.69 (3.97) ^b^	6.75 (1.49) ^a^	9.06 (1.04) ^a^	1.31 (0.48) ^a^	0.24 (0.12) ^a^
**Rootstock**								
*Citrus macrophylla*	163.25 (44.01) ^a^	62.60 (5.76) ^ab^	94.26 (10.97) ^a^	15.01 (4.23) ^a^	6.74 (1.27) ^a^	9.15 (1.14) ^a^	1.36 (0.69) ^a^	0.26 (0.08) ^a^
*citrus aurantium*	176.13 (45.14) ^b^	64.07 (5.67) ^b^	98.05 (12.46) ^b^	16.90 (5.23) ^b^	6.83 (1.70) ^a^	9.02 (1.83) ^a^	2.31 (2.46) ^b^	0.27 (0.11) ^a^
*Citrus aurantium/Citrus sinensis*	159.25 (35.92) ^a^	61.96 (4.57) ^a^	94.37 (10.53) ^a^	16.51 (4.10) ^b^	6.97 (1.41) ^a^	9.08 (1.13) ^a^	1.35 (0.62) ^a^	0.25 (0.08) ^a^

The different letters within the columns for each independent variable (type of substrate and rootstock) indicate significant differences according to the Tukey HSD test (*p* < 0.05).

**Table 4:** Peel and juice color variations of ‘Verna’ lemons obtained on different rootstocks and substrates, where L* represents luminosity, a* green/red, b* blue/yellow, C* chroma values, H° the hue angle, and CI the citrus color index. The values represented are the mean with their standard deviation in parentheses.

	L*	a*	b*	C*	H°	CI
**External color**						
**Substrate (sediment content, %)**						
25%	70.12 (3.90) ^a^	4.90 (3.04) ^a^	53.02 (4.17) ^a^	53.32 (4.83) ^a^	84.92 (3.24) ^c^	1.29 (0.74) ^a^
50%	71.62 (2.69) ^b^	7.45 (2.59) ^b^	56.54 (3.41) ^b^	57.08 (3.57) ^b^	82.56 (2.43) ^a^	2.02 (0.59) ^b^
75%	71.86 (2.34) ^b^	6.82 (2.57) ^b^	57.17 (2.74) ^b^	57.62 (2.89) ^b^	83.26 (2.48) ^b^	1.28 (0.68) ^a^
**Rootstock**						
*Citrus macrophylla*	70.80 (3.54) ^a^	6.22 (2.95) ^a^	55.24 (4.19) ^a^	55.66 (4.33) ^a^	83.74 (2.97) ^a^	1.48 (0.60) ^a^
*Citrus aurantium*	71.70 (2.75) ^b^	6.61 (2.72) ^a^	54.93 (4.08) ^a^	55.38 (4.24) ^a^	83.23 (2.60) ^a^	2.38 (0.35) ^b^
*Citrus aurantium/Citrus sinensis*	71.18 (3.05) ^ab^	6.48 (3.14) ^a^	55.55 (4.40) ^a^	56.00 (4.57) ^a^	83.51 (3.10) ^a^	1.62 (0.83) ^a^
**Internal color**						
**Substrate (sediment content, %)**						
25% silt	42.02 (1.86) ^b^	−067 (0.23) ^a^	2.08 (0.68) ^a^	2.21 (0.63) ^a^	109.48 (9.79) ^b^	−8.85 (5.85) ^a^
50% sediment	41.82 (1.47) ^b^	−0.56 (0.36) ^a^	2.36 (0.69) ^b^	2.45 (0.72) ^a^	102.73 (8.88) ^a^	−5.55 (4.12) ^b^
75% sediment	41.21 (0.93) ^a^	−0.55 (0.31) ^a^	2.71 (0.58) ^c^	2.78 (0.61) ^b^	100.77 (5.70) ^a^	−4.67 (2.50) ^b^
**Rootstock**						
*Citrus macrophylla*	41.75 (1.61) ^a^	−0.52 (0.29) ^b^	2.30 (0.70) ^a^	2.38 (0.68) ^a^	102.72 (9.38) ^a^	−5.54 (4.27) ^b^
*Citrus aurantium*	41.65 (1.10) ^a^	0.80 (0.28) ^a^	2.57 (0.68) ^b^	2.70 (0.72) ^b^	107.32 (3.82) ^b^	−7.49 (1.66) ^a^
*Citrus aurantium/Citrus sinensis*	41.75 (1.74) ^a^	−0.54 (0.29) ^b^	2.25 (0.70) ^a^	2.35 (0.65) ^a^	104.48 (11.43) ^ab^	−6.72 (6.64) ^ab^

The different letters within the columns for each independent variable (type of substrate and rootstock) indicate significant differences according to the Tukey HSD test (*p* < 0.05).

**Table 5:** Variation of pH, soluble solids (TSS, °Brix), titratable acidity (TA, g citric acid L^−1^), and maturity index (MI) parameters of ‘Verna’ lemons obtained on different rootstocks and substrates. The values represented are the mean with its standard deviation in parentheses.

	pH	TSS (°Brix)	TA (g Citric Acid L^−1^)	MI
**Substrate (sediment content, %)**				
25%	2.54 (0.50) ^a^	8.96 (0.31) ^a^	59.71 (3.56) ^a^	1.50 (0.06) ^a^
50%	3.04 (0.54) ^a^	9.10 (0.55) ^b^	62.79 (3.03) ^ab^	1.45 (0.09) ^ab^
75%	2.49 (0.51) ^b^	9.02 (0.56) ^b^	64.32 (6.05) ^b^	1.41 (0.10) ^b^
**Rootstock**				
*Citrus macrophylla*	2.54 (0.50) ^a^	8.96 (0.38) ^a^	62.33 (2.86) ^ab^	1.44 (0.07) ^a^
*Citrus aurantium*	3.04 (0.54) ^b^	9.10 (0.69) ^a^	59.75 (6.23) ^a^	1.52 (0.07) ^b^
*Citrus aurantium/Citrus sinensis*	2.49 (0.51) ^a^	9.04 (0.36) ^a^	63.88 (3.93) ^b^	1.42 (0.09) ^a^

The different letters within the columns for each independent variable (type of substrate and rootstock) indicate significant differences according to the Tukey HSD test (*p* < 0.05).

**Table 6:** Total phenolic content (TPC, mg GAE 100 mL^−1^) and antioxidant capacity variations measured according to ABTS, DPPH, and FRAP (mmol Trolox L^−1^) for ‘Verna’ lemons obtained on different rootstocks and substrates. The values represented are the mean with its standard deviation in parentheses.

	ABTS (mmol Trolox L^−1^)	DPPH (mmol Trolox L^−1^)	FRAP (mmol Trolox L^−1^)	TPC (mg GAE 100 mL^−1^)
**Substrate (sediment content, %)**				
25%	1.49 (0.11) ^a^	3.08 (0.20) ^a^	1.08 (0.11) ^a^	93.63 (19.08) ^a^
50%	1.51 (0.18) ^a^	3.10 (0.31) ^a^	1.14 (0.13) ^ab^	83.49 (10.65) ^a^
75%	1.58 (0.22) ^a^	3.13 (0.15) ^a^	1.25 (0.21) ^b^	93.16 (22.85) ^a^
**Rootstock**				
*Citrus macrophylla*	1.48 (0.14) ^a^	3.25 (0.10) ^b^	1.05 (0.14) ^a^	80.77 (8.96) ^a^
*Citrus aurantium*	1.61 (0.23) ^a^	3.12 (0.18) ^b^	1.16 (0.13) ^ab^	104.80 (19.89) ^b^
*Citrus aurantium/Citrus sinensis*	1.49 (0.12) ^a^	2.91 (0.24) ^a^	1.25 (0.15) ^b^	87.69 (17.01) ^a^

The different letters within the columns for each independent variable (type of substrate and rootstock) indicate significant differences according to the Tukey HSD test (*p* < 0.05).

**Table 7:** Sugars and organic acids (g 100 mL^−1^) identified in the juice of ‘Verna’ lemon grown with different rootstocks and substrates. The results represent the mean values (*n* = 5) and their standard deviation in parentheses.

	Glucose (g 100 mL^−1^)	Fructose (g 100 mL^−1^)	Citric Acid (g 100 mL^−1^)	Malic Acid (g 100 mL^−1^)	Ascorbic Acid (g 100 mL^−1^)	Succinic Acid (g 100 mL^−1^)
**Substrate (sediment content, %)**						
25%	0.75 (0.05) ^a^	0.58 (0.04) ^a^	4.34 (0.19) ^a^	0.43 (0.08) ^a^	0.06(0. 00) ^a^	0.59 (0.06) ^a^
50%	0.79 (0.03) ^a^	0.55 (0.05) ^a^	4.41 (0.17) ^a^	0.41 (0.06) ^a^	0.06(0. 00) ^a^	0.61 (0.16) ^a^
75%	0.79 (0.06) ^a^	0.55 (0.06) ^a^	4.46 (0.22) ^a^	0.38 (0.08) ^a^	0.06(0. 00) ^a^	0.61 (0.12) ^a^
**Rootstock**						
*Citrus macrophylla*	0.77 (0.03) ^a^	0.57 (0.02) ^a^	4.40 (0.13) ^a^	0.42 (0.07) ^b^	0.06(0. 00) ^a^	0.57 (0.09) ^a^
*Citrus aurantium*	0.76 (0.07) ^a^	0.54 (0.09) ^a^	0.32 (0.29) ^a^	0.35 (0.07) ^a^	0.06 (0.00) ^b^	0.66 (0.18) ^a^
*Citrus aurantium/Citrus sinensis*	0.78 (0.04) ^a^	0.58 (0.01) ^a^	4.46 (0.14) ^a^	0.44 (0.03) ^b^	0.06 (0.00) ^ab^	0.59 (0.08) ^a^

The different letters within the columns for each independent variable (type of substrate and rootstock) indicate significant differences according to the Tukey HSD test (*p* < 0.05).

## Data Availability

Data are contained within the article.
